# Mass Media Beauty Standards, Body Surveillance, and Relationship Satisfaction within Romantic Couples

**DOI:** 10.3390/ijerph19073833

**Published:** 2022-03-23

**Authors:** Chiara Rollero

**Affiliations:** Department of Psychology, University of Turin, 10124 Torino, Italy; chiara.rollero@unito.it; Tel.: +39-0116702534

**Keywords:** objectification processes, body surveillance, mass media, relationship satisfaction, psychosocial perspective

## Abstract

As part of objectification processes, individuals engage in body surveillance, whereby they constantly assess the extent to which their external appearance conforms to culturally valued ideals. Mass media play a key role in fostering the objectification and internalization of media beauty standards and increases body surveillance. At the individual level, the literature has largely demonstrated that body surveillance leads to a variety of negative psychological outcomes, but little research has focused on the consequences of body surveillance in the context of romantic relationships. Using dyadic data from couples who identified as heterosexual, the present study examined relations among internalization of media standards, body surveillance, surveillance of the partner’s body, surveillance from the partner, and relationship satisfaction. There were 438 participants (219 couples) recruited using snowball sampling. They were surveyed with an anonymous online questionnaire. Results showed that internalization of media standards was related to body surveillance in both men and women, and to surveillance of the partner’s body and relationship satisfaction in men only. For both sexes, surveillance of the partner’s body was negatively associated with relationship satisfaction. For women only, surveillance from the partner was also negatively related to relationship satisfaction. Implications are discussed.

## 1. Introduction

Objectification theory [1] suggests that women in Western society are perceived as sexual objects and a collection of body parts whose value is determined by their physical appearance and sexual attractiveness. As a consequence, women internalize this third-person perspective, valuing the observable characteristics of their body more than the internal, unobservable characteristics. As part of this process, women engage in body surveillance, whereby they constantly assess the extent to which their external appearance is in line with culturally valued ideals [1,2].

One of the most relevant socialization agents promoting objectification and body surveillance is mainstream media, whose content frequently equates a person’s worth with their sexual attractiveness [2]. The power of these representations in triggering body surveillance stems from both their prevalence and accessibility [3]. However, it is not just exposure to mass media per se that proves detrimental: the real problem arises when individuals internalize such sexually objectifying messages [4,5]. Internalizing objectifying messages from the media leads people to surveil their bodies and guides the perception of their worth [5,6]. Empirical research has largely shown that internalization of beauty ideals in the media has a direct impact on self-objectification processes and body surveillance for both adolescents and adults, e.g., [3,7,8,9,10].

At individual level, research has largely demonstrated that body surveillance leads to a plethora of negative outcomes. It increases body shame and appearance anxiety, decreases awareness of internal bodily states, and reduces the likelihood of being in the creative and pleasurable state of “flow” (for an overview, see [1,11,12]). Furthermore, body surveillance is associated with eating disorders, depression, sexual dysfunction, increased fear of rape, and decreased self-esteem, i.e., [13,14,15,16,17,18].

Although objectification theory was developed in relation to women’s experiences, research has explored the applicability of this framework to investigate men’s experiences as well. In general, men seem to show lower body surveillance than women [19], but male adults are becoming progressively more worried about their physical appearance [10,20,21]. This appears to be related to the growing tendency to objectify men’s bodies in Western culture, which increases body image concerns among men [22,23]. Consistent with findings on women, body surveillance in men correlates with lower self-esteem, negative mood, poorer health perceptions, and eating disorders [16,24,25]. Moreover, self-objectification processes have been implicated in explaining drive for muscularity, excessive exercise, and steroid use [26,27]. In summary, a large number of studies based on objectification theory have demonstrated associations between body surveillance processes and relevant psychological outcomes in both female and male populations.

**Figure 1:**
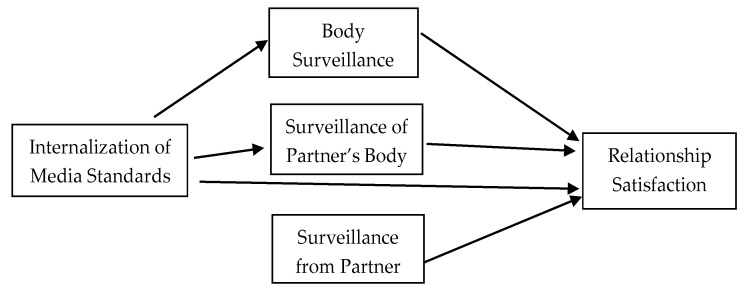
The hypothesized model.

Empirical studies on body surveillance and objectification processes abound, but relatively little research has addressed these issues in the context of romantic relationships. According to Ramsey and colleagues [28], this is a curious omission, given that physical appearance is considered a major factor in romantic attraction, and the conceptualization of objectification tends to overemphasize physical appearance. The few studies applying objectification theory to the context of romantic relationships suggest that the partner may play a crucial role in mitigating or exacerbating appearance concerns [29,30]. In addition, Mahar and colleagues [31] found that both men and women who had higher scores on their partners’ body surveillance had lower relationship commitment and relationship satisfaction. Accordingly, individuals who are objectified by their romantic partner might experience distress in their romantic relationship [28,32]. The purpose of the present study was to extend existing knowledge in objectification research by using dyadic data from heterosexual couples to examine the following: (1) whether the internalization of appearance media standards is related not only to one’s own body surveillance, but also to surveillance of the partner’s body and relationship satisfaction; and (2) whether body surveillance, surveillance of the partner’s body, and surveillance from the partner are related to relationship satisfaction (Figure 1). Finally, in line with literature recommendations [14,33], rather than assuming equivalence among these constructs in both sexes, I examined these hypotheses separately in males and females.

## 2. Materials and Methods

### 2.1. Participants and Procedure

The study enrolled 438 participants (219 couples) currently in heterosexual relationships. Their ages ranged from 18 to 41 years (M = 22.38, SD = 3.71). They were all Caucasian. Relationship duration ranged from 5 to 192 months (M = 31.21 months, SD = 31.49). Participants were recruited using snowball sampling beginning with online postings by the researcher and her students. The postings were published on the students’ social media (i.e., Facebook, Instagram, Twitter, and WhatsApp) and on the researcher’s personal academic website. The link from the posting directed participants to a secure, anonymous online questionnaire (Google Form) where they read an informed consent form before beginning the study. Each participant was also informed that their participation was voluntary, and that they could discontinue the study at any time. The questionnaire took approximately 20 min to complete. Participants completed the survey on their computers. No compensation was given for participation. Following Strelan and Pagoudis [34], to match couples and ensure anonymity, the opening question of the survey asked the first participating partner in a couple to create a codename. He/she was required to note the code and communicate it to the partner, who subsequently reported the same code when he/she completed the survey.

The Ethics Committee of the University of Turin, Italy, approved the study protocol (Ethical approval code: 131118).

### 2.2. Measures

The questionnaire included the following measures, whose reliability was established in previous studies, e.g., [32,35,36,37]:

1. Internalization of media beauty standards: The 9-item Internalization-General Subscale of the Sociocultural Attitudes Towards Appearance Questionnaire-3 (SATAQ-3) [35] was used. Items were rated on a 5-point Likert scale (1 = definitely disagree, 5 = definitely agree) (for the current study, α = 0.94).
2. Body surveillance: The 8-item Body Surveillance Subscale of McKinley and Hyde’s [36] Objectified Body Consciousness Scale was used. Responses were measured on a 1 (=definitely disagree) to 7 (=definitely agree) Likert-type scale (for the current study, α = 0.79).
3. Surveillance of the partner’s body: the Body Surveillance Subscale of the Objectified Body Consciousness Scale was used, rewording items so that all references to “I”, “my body”, or “how I look” instead referred to “my partner”, “my partner’s body”, and “how my partner looks” [32] (for the current study, α = 0.69).
4. Surveillance from the partner: for each member of the couple, the responses given by the other member to the above reported surveillance of the partner’s body measure were used.
5. Relationship satisfaction: The 7-item Relationship Assessment Scale [37] was used to measure global relationship satisfaction. Items were rated on 5-point Likert scale (for the current study, α = 0.88).

Finally, a list of socio-demographic items was included, assessing sex, age, ethnicity, and duration of the current romantic relationship.

## 3. Results

### 3.1. Bivariate Analyses

Bivariate analyses were performed using SPSS 27. Correlations among the variables are provided in Table 1.

Student’s *t* tests comparing men and women’s scores across the variables are reported in Table 2. Women reported higher levels of internalization of media standards and body surveillance categories, whereas men outscored women on surveillance of the partner’s body. No significant sex differences emerged concerning relationship satisfaction.

### 3.2. Testing the Hypothesized Model

The hypothesized paths among variables were tested via structural equation modelling using AMOS 27. All variables in the model were treated as observed variables, and the covariance matrices were analyzed using maximum likelihood estimation procedures. To investigate the moderating role of sex, sex invariance of paths was tested using multigroup moderation analysis. The following steps were conducted: (a) an unconstrained multigroup model across sex was examined in which the same pattern of structural paths was tested without constraints across groups; and (b) a constrained multigroup model was examined, where structural paths were constrained to be equal across groups. As suggested [38], the model fit was tested by using different fit indexes to reduce the impact of their limits. Specifically, the following were used as criteria for acceptable model fit: χ^2^, confirmatory fit index (CFI), normed fit index (NFI), incremental fit index (IFI), and root mean square error of approximation (RMSEA). For CFI, NFI, and IFI, values higher than 0.90 are judged as satisfactory. As for RMSEA, values lower than 0.08 are judged as satisfactory. Additionally, the Akaike information criterion (AIC) was used to compare the unconstrained and the constrained model. The absolute AIC value is irrelevant, but the models that generate lower AIC values are better fitting [38].

Analysis of the unconstrained multigroup model indicated this model fit the data adequately: χ^2^ = 5.76, n.s.; χ^2^/gdl = 2.88; CFI = 0.98; NFI = 0.97; IFI = 0.98; RMSEA = 0.06. The constrained multigroup model showed good fit indexes as well: χ^2^ = 19.15, *p* < 0.05; χ^2^/gdl = 2.39; CFI = 0.93; NFI = 0.90; IFI = 0.94; RMSEA = 0.06. However, AIC indicated that the unconstrained model (AIC = 81.75) provided a better fit to the data than the constrained one (AIC = 83.15). The significant Chi-square difference (Δχ^2^ = 13.39, Δdf = 6, *p* < 0.05) indicated that one or more structural paths were different across sex. When this statistic is significant, the model with the smaller Chi-square is chosen [39]. Thus, the baseline model was selected as the final model.

**Figure 2:**
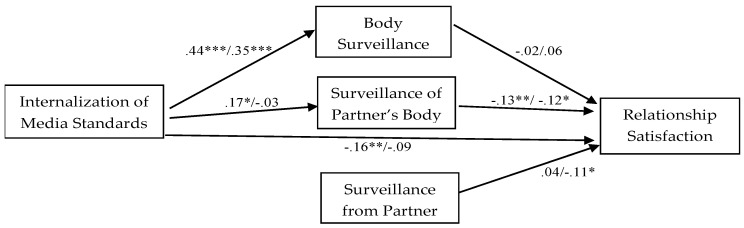
Standardized path coefficients of the hypothesized model for men and women, respectively. * *p* < 0.05; ** *p* < 0.01; *** *p* < 0.001.

The final model accounted for 36% of the variance in the relationship satisfaction category for men and 37% for women. Most of the paths proved significant in the hypothesized models at least for one sex, except for the path from body surveillance to relationship satisfaction, which was not significant for either men or women. By testing for structural invariance, I was able to determine how each path coefficient differed by sex. The internalization of media standards was associated to body surveillance in both groups (men β = 0.44, *p* < 0.001; women β = 0.35, *p* < 0.001), and for men only it was positively related to surveillance of the partner’s body (β = 0.17, *p* < 0.05) and negatively to relationship satisfaction (β = −0.16, *p* < 0.01). For both men and women, surveillance of the partner’s body was negatively associated with relationship satisfaction (men β = −0.13, *p* < 0.01; women β = −0.12, *p* < 0.05), whereas for women, surveillance from the partner was negatively associated with relationship satisfaction (β = −0.11, *p* < 0.05). Finally, in both sexes, body surveillance and surveillance of the partner’s body were correlated (men r = 0.26, *p* < 0.01; women r = 0.24, *p* < 0.01), as well as surveillance of the partner’s body and surveillance from the partner (men r = 0.20, *p* < 0.01; women r = 0.22, *p* < 0.01). The standardized coefficients for men and women are reported in Figure 2.

Multigroup moderation analysis was conducted using a sequential constraints approach in order to test whether paths were significantly variant by sex. Individual pathways were tested for invariance by successively constraining each path to be equal across groups to locate the variances in the model. Corresponding Chi-square difference tests were then used to determine whether sex significantly moderated the paths. Results showed that sex moderated the relationships between internalization of media standards and surveillance of the partner’s body, Δχ^2^ (1) = 4.62, *p* < 0.05, as well as surveillance from the partner and relationship satisfaction, Δχ^2^ (1) = 5.08, *p* < 0.05.

## 4. Discussion

The previous literature on objectification processes and body surveillance has focused on the individual level. The present study extended previous knowledge by examining these constructs among romantic heterosexual couples.

Although there was no sex difference in relationship satisfaction, consistent with previous studies [1,19], women showed higher internalization and body surveillance, whereas men reported higher levels of partner objectification than women.

The results confirmed that the internalization of media appearance standards promotes body surveillance in both sexes. When viewing mass media that objectify people, men and women internalize the message that their worth should be based on their physical body, which in turn leads to constant body monitoring, to assess whether their external appearance meets culturally valued ideals [4,5,6].

For men only, internalization was related to the surveillance of the partner’s body and was associated with lower relationship satisfaction. This could be due to a different emphasis on the male versus female body in the media. Even though there is a considerable number of objectified male models in today’s mass media, exploitative and sexualized portrayals of women are more common and widespread [5,40]. This likely leads men to value their partner’s appearance over her competence more than women. Consistent with Social Comparison Theory [41], men may compare their partner’s body to the ‘perfect’ bodies portrayed in the media and feel dissatisfied because of this upward comparison [16].

Furthermore, in our data the surveillance of the partner’s body were associated with lower relationship satisfaction in both men and women. Because physical attractiveness plays an essential role in romantic relationships, appearance is usually a focal point. However, our results show that focusing on a partner’s appearance to the extent that his/her nonphysical qualities are devalued (e.g., through chronic body surveillance) has negative effects on relationship satisfaction. Notably, while previous research has focused primarily on the consequences of being objectified, this study presents a possible outcome of perpetrating objectification processes. In other words, body surveillance appears to have negative consequences for both the target and the perpetrator.

Finally, only in women, was body surveillance from the partner related to lower relationship satisfaction. Again, this could be due to the fact that objectification processes are still more prevalent in women than in their male counterpart. A woman perceiving that her romantic partner objectifies her may feel an imbalance in the relationship, leading to lower relationship satisfaction [28]. In addition, body surveillance from the partner may come at the expense of the emotional intimacy of the relationship, as appearance is prioritized over all other personal characteristics.

Taken together, the current findings offer new insights into objectification processes, and in particular on the relationship between the studied categories—internalization of media standards, body surveillance, and relationship satisfaction—by using dyadic data from a relatively large sample of romantic couples. An additional strength of the present study is that participants responded based on their current relationship, rather than hypothetical or past relationships, implying that the variables considered have potential real-world consequences. In addition, the dyadic design allowed for the examination of both actor and partner effects.

The present paper has several limitations that provide directions for future research. The first relates to the level of relationship commitment, which was not considered in the current study. One possibility is to investigate the difference between a loving, supportive relationship and casual sexual encounters. It seems plausible that body surveillance and surveillance of the partner’s body might have a differential impact on satisfaction depending on the type of relationship commitment. Another limitation includes the socio-demographic characteristics of the participants, who were Caucasian, heterosexual, and mainly young adults. For future studies, it may be important to include people of different ethnicities, and, in line with the previous limitation, couples of different sexual orientations and ages who had relationships of different lengths. As Buss and Schmitt have suggested [42,43], men and women can use different partner selection strategies in short- versus long-term mating contexts. Finally, self-reported measures may have been affected by social desirability, as participants may be reluctant to reveal personal aspects of their relationship, especially if they perceive them to be unflattering.

## 5. Conclusions

This study highlights the importance of examining both self- and partner body surveillance in the context of romantic heterosexual relationships. Our general finding was that surveillance of the partner’s body, and for women, surveillance from the partner, were associated with lower relationship satisfaction. In addition, for men, internalization of mass media beauty standards was related to higher levels of partner’s body surveillance. The implication is that objectification processes, in the media and elsewhere, are involved in a wide range of negative outcomes and that the internalization of these objectifying messages is likely to be harmful even to intimate romantic relationships. In line with the assumption of Strelan and Pagoudis [34], it is confirmed that objectification is a self-perpetuating interpersonal process, and the current work points to the various ways in which objectification and body surveillance can permeate the lives of men women, especially those in intimate relationships.

## Figures and Tables

**Table 1:** Pearson’s correlations between variables.

	2.	3.	4.	5.	6.	7.	8.
1. Women’s Internalization of Media Standards	0.23 **	0.38 **	0.19 *	−0.00	0.11	−0.12	−0.19 *
2. Men’s Internalization of Media Standards		−0.05	0.39 **	0.12	0.19 *	−0.08	−0.26 **
3. Women’s Body Surveillance			0.19 *	0.29 **	0.05	−0.02	−0.05
4. Men’s Body Surveillance				0.17	0.36 **	0.02	−0.17
5. Women’s Surveillance of the Partner’s Body					0.28 **	−0.19 *	−0.02
6. Men’s Surveillance of the Partner’s Body						−0.21 **	−0.22 **
7. Women’s Relationship Satisfaction							0.46 **
8. Men’s Relationship Satisfaction							

** *p* < 0.001; * *p* < 0.01.

**Table 2:** Sex differences on the study variables: means, standard deviations, and Student’s *t*-Test scores.

		Mean	SD	*t*
Internalization of Media Standards	Men	1.78	0.85	7.39 **
	Women	2.35	0.98	
Body Surveillance	Men	3.73	1.03	8.35 **
	Women	4.46	0.96	
Surveillance of the Partner’s Body	Men	3.55	0.89	−2.51 *
	Women	3.37	0.90	
Relationship Satisfaction	Men	4.27	0.63	−0.65
	Women	4.24	0.64	

** *p* < 0.01; * *p* < 0.05.

## Data Availability

The data presented in this study are available on request from the author. The data are not publicly available due to ethical restrictions.
